# Acknowledging existential moments and meaning-making in palliative care: A hermeneutic study of physicians’ experience

**DOI:** 10.1017/S1478951525100230

**Published:** 2025-06-20

**Authors:** Martina Ann Kelly, Ellen McLeod

**Affiliations:** 1 Department of Family Medicine, Cumming School of Medicine, G329, Health Sciences Centre, University of Calgary, Alberta, Canada; 2 Department of Oncology, Palliative Care & End of Life, Cumming School of Medicine, University of Calgary, Alberta, Canada

**Keywords:** palliative care, qualitative research, hermeneutics, job satisfaction, burnout, compassion fatigue, reward, existential

## Abstract

**Objective.** Witnessing existential suffering in patients is associated with increased risks of compassion fatigue and burnout in palliative care physicians. Finding meaning is a protective factor for burnout and is associated with increased job satisfaction and a sense of purpose.

While the significance of finding meaning through the doctor-patient relationship is widely recognized, less is known about the subjective experience of palliative care physicians, how they ascribe meaning, and how meaning sustains them. The aim of this study was to describe and interpret how palliative care physicians experience meaning when caring for patients.

**Method.** Hermeneutic-phenomenology, inspired by the philosophy of Heidegger and Gadamer, informed the methodological approach. Ten palliative care physicians, caring for adult patients, completed semi-structured interviews. Van Manen’s “lifeworld existentials” supported our reflexive hermeneutic analysis to interpret participants’ moments of meaning-making.

**Results.** Our analysis identified two interpretive concepts for meaning-making: moments of connection and moments of transformation. Meaningful connection occurred when physicians and patients together acknowledged existential suffering in death and dying and experienced it on a personal, human level. Often, experiences were fleeting but had a lasting impact. Experiences of connection had a transformational effect on physicians and were associated with a sense of reward and purpose in palliative care work.

**Significance of results.** Findings are discussed in relation to philosophical literature on the experience of time, contrasting man-made time with the existential experience of time. Moments of connection and transformation experienced by palliative care physicians fueled their commitment to their profession. At a time when burn-out is rife, identifying, describing, and understanding moments of meaning may offer protective benefits for physicians working in palliative care.

## INTRODUCTION

Palliative care physicians work to relieve the suffering of death in all its forms: physical, psychological, social, and existential (Connor and Sepulveda 2014). Existential suffering is a particularly poignant aspect of palliative care: when faced with death, patients may wrestle with an “existential slap,” where the reality and inevitability of one’s mortality assume new significance (Coyle 2004).

Bearing witness to – and caring for – people during this time can take a toll on physicians (Back et al. 2016; Fillion and Vachon 2018; Vachon and Guité-Verret 2020). A growing body of evidence shows how continual exposure to suffering, loss, and death can erode the long-term health and well-being of palliative care doctors, resulting in compassion fatigue and burnout (Cherny et al. 2015; Galiana et al. 2022; Kase et al. 2019; Kavalieratos et al. 2017; Slocum-Gori et al. 2013). The costs of burnout to patients, physicians, and society include increased patient harm, loss of physician workforce, poor mental health, and suicide among physicians (Dewa et al. 2014; West et al. 2018; Williams et al. 2020). Mitigating these costs, and stemming the epidemic of burnout, has recently become a major focus of health care policy and education (Haverfield et al. 2020; Sikka et al. 2015; Toubassi et al. 2023).

Interventions to counter burnout have been broadly categorized as institutional, organizational, and existential (Abedini et al. 2018; Engebretsen and Bjorbækmo 2020). Existential burnout refers to the personal loss of coherence and meaning in clinical work. In contrast, finding meaning by attending to individual, subjective experiences of meaning-making is associated with higher self-reported job satisfaction (West et al. 2018), improved self-reported health (Steger and Kashdan 2009) and increased quality of life (Krause 2009). Some research suggests that identifying and supporting internal motivators, such as meaning, may be more effective than some extrinsic motivators at supporting physician wellness and may be more enduring in enhancing satisfaction (Deci and Ryan, n.d.; Pink 2011). One important source of meaning-making in medicine is time spent with patients (Branch et al. 2017; Hipp et al. 2017; Serwint and Stewart 2019).

Meaning in medicine arises through interpersonal interactions and the nurturing power of doctor-patient relationships (Haverfield et al. 2020). Meaning is not determined by situations per se, but rather by the meaning we attribute to situations (Beck et al. 1979). To date, while the significance of finding meaning through the doctor-patient relationship is acknowledged, less is known about the subjective experience of how physicians – including those in palliative care – ascribe meaning or how meaning sustains them. Developing such an understanding, we surmised, could be a valuable resource to positively support palliative care physicians as they grapple with the day-to-day realities of dealing with existential suffering, including their own. This study aimed to describe and interpret what gives meaning to physicians when caring for patients with palliative care needs. Our research question was, “How do palliative care physicians experience meaning when caring for patients with palliative care needs?”

## METHODS

### Theoretical framework

Hermeneutic phenomenology aims to understand how something has been experienced and understood (Harris 2017; Moules 2015). Phenomenology seeks to bring experiences to the fore and is described as attending to “lived” experience. Hermeneutic phenomenology draws historically on hermeneutics, the practice of interpreting texts. Heidegger (2010) and Gadamer (Gadamer 2013) advanced ideas of interpretation in phenomenology to emphasize that, as people, we constantly interpret and make sense of our lived experiences. The goal of hermeneutics is not to simplify, reduce, or categorize data (as in thematic analysis), but rather to draw attention to multiple meanings. This process draws the reader to new understandings and insights (Moules 2015). As such, hermeneutics fits within a constructivist-interpretive framework, where meaning is construed through a dynamic interplay between data immersion, analysis, and researcher reflexivity.

### Ethical considerations

This study received ethical approval from the Conjoint Health Research Ethics Board (CHREB), University of Calgary (Ethics ID: REB22-0998). All participants provided written, informed consent. Ethical considerations included the possibility that participants might experience distress while recalling emotionally intense experiences. For this reason, the interview guide was flexible, and participants were given time to pause or stop the interview as needed. They were also offered a debrief conversation if required, although no participants requested this follow-up support.

### Setting and participants

The study setting was a palliative and end-of-life care service for adult patients across all care settings in Western Canada. All palliative care physicians affiliated with the service were eligible to participate.

### Sampling and recruitment

Hermeneutic-phenomenological studies typically require small sample sizes with the aim of collecting in-depth, rich data (Moules 2015). Purposive sampling was used to identify physicians with a range of clinical experience, including location of work (hospital, community, and hospice settings) and years in clinical practice. An email outlining the study was sent to eligible participants by the department's medical director, and a reminder was sent 1 month later. Interested participants contacted E.M., who provided further study information and obtained informed consent. As a token of appreciation, participants were offered a $25 gift card.

### Data collection

We devised a semi-structured interview guide, which invited participants to describe in detail an experience of meaning in relation to their care of patients with palliative care needs. We defined an experience of meaning as “moments that provide you a sense of meaning, satisfaction, or fulfillment, in your work.” To help participants reflect on their experience, we used prompts to draw out details, based on Van Manen’s lifeworld “existentials,” which describe key facets of how we experience something through time, body, space, and relationships (van Manen M 2023). The interview guide was piloted with two palliative care colleagues, and no changes were suggested (Table 1 details the interview guide). Interviews were held at a time and location convenient for participants. All interviews were conducted by E.M. between October 2022 and March 2023. Interviews lasted 20–67 minutes, with a mean duration of 35 minutes. Following the interview, E.M. transcribed the audio-recorded interview verbatim, noting pauses, tone, and emotional expressions such as sighs or tearfulness. Field notes were recorded following interviews and used as an *aide-memoire* during analysis. Any possible identifying information was removed, and interviews were allocated a number. Data collection and analysis were iterative, and data collection ceased when sufficient data provided rich, detailed accounts of participants’ experiences.

**Table 1.** Qualitative interview guide

Questions
Describe a meaningful encounter in as much detail as you can (probe using experiences of time, body, space, and relationships).
What was meaningful about that encounter for you?
What made you think of that encounter specifically?
When did you know that interaction was so meaningful for you?
Has your perspective on this encounter changed over time?
Reflecting on this encounter, which components give you the greatest sense of meaning?
Are there any other interactions that have stuck with you in a similar way? (repeat Q1)
In reflecting on these encounters, is there anything important you’d like to add?

### Analysis

**Figure 1.**
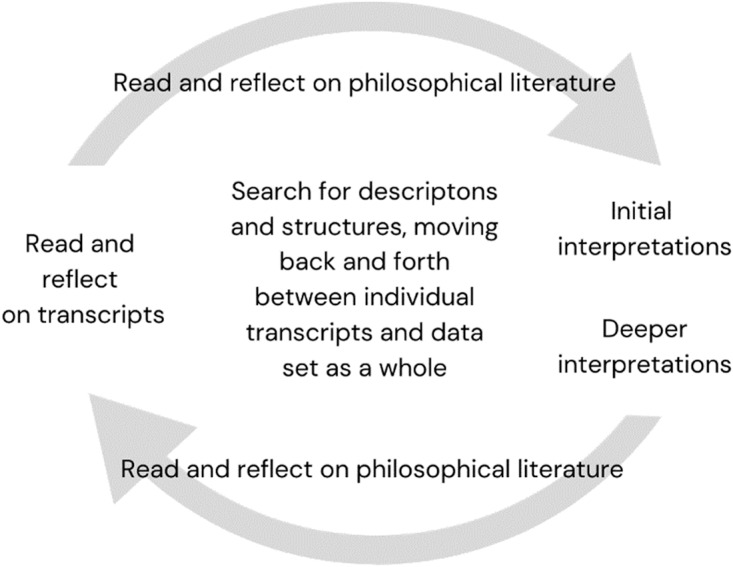
Hermeneutic analytic process (informed by Crowther and Thomson 2020, 2022).

Analysis comprised three intersecting steps (Crowther and Thomson 2020, 2022) (see Fig. 1), supported by regular meetings between M.K. and E.M.

Description: Transcripts were read independently by the authors. Each noted initial thoughts on the margins of the transcript. Experiences that physicians identified as meaningful accounts were extracted from each interview. Descriptive features were identified, aided by Van Manen’s existentials (van Manen 2023). “Story-crafting” was used to bring the phenomenon into sharper relief by focusing on phrases and meaning units that preserved the experience (Crowther et al. 2017). An example of a crafted story of a meaningful moment is provided in Box 1. Each transcript was examined individually before attending to patterns across the dataset.

Reflection: To develop our reflexive analysis, we questioned and reflected on our interpretations through team meetings, writing reflective memos, and moving back and forth between transcripts and notes to develop an initial understanding of the significant and important aspects of meaning-making. At the time of the study, E.M. was a palliative care resident with previous work experience in family medicine and social work. M.K. is an academic family physician who provides palliative care to her patients. Both authors have experience in qualitative research (MSc, PhD). Together, we developed our understanding of the significant aspects of clinicians’ meaning-making in palliative care, including reflections on our experiences as women, physicians, wives, mothers, and our personal and professional experiences with death and dying.

Reflexivity is key to Gadamer’s idea of “historicity” (Gadamer 2013), which refers to how understanding is informed by our history and traditions of experience; probing these understandings helps us become aware of how our prejudices, or pre-judgments, inform our interpretations, whilst also enabling us to become aware of new ways of interpreting and understanding the phenomenon under scrutiny (Harris 2017).

Interpretation: The final interpretation was deepened by reading existential phenomenological literature, drawing on ideas from Heidegger (Heidegger 2010) and Emanuel Levinas (Levinas 1979). Hermeneutic research does not generate universal or generalizable truths, nor definitive or final accounts; rather, it draws attention to the meaningful possibilities that surface through attention (Moules et al. 2012).

Rigor in hermeneutic phenomenology relies on principles of rich description, which address a reader so they can relate to the material presented, inducing a sense of connection and wonder, affording new insights into life-meaning or professional practice (Moules 2015; van Manen 2023). To ensure credibility and resonance, findings were presented through several faculty presentations, which endorsed their relevance.

## Results

### Participants

Ten physicians were interviewed between October 2022 and March 2023 (see Table 2). Interviews lasted 20–67 minutes, with a mean duration of 33 minutes.

**Table 2.** Demographic details of participants (*N* = 10)

	Participants (*n*) (%)
Sex	
Female	4 (40)
Male	6 (60)
Age (years)	
≤35	3 (30)
36−49	2 (20)
≥50	5 (50)
Years in practice	
≤10	4 (40)
11−20	3 (30)
≥21	3 (30)

### Interpretative findings

The overarching finding of our analysis is captured by the phrase “acknowledging existential moments.” Two interpretive concepts helped us understand what made these moments meaningful: moments of connection and moments of transformation.

#### Acknowledging existential moments

Moments of meaning were characterized by a fleeting but profound sense of connection, in which physicians and patients acknowledged mortality and death. In these moments, physicians described experiencing a sense of humanity and shared vulnerability with their patients (see an example in Box 1 and Appendix 1). Meaningful moments provided physicians with personal insight. They affirmed their professional identity, confirmed their role as palliative care physicians, and inspired them to seek out similar moments.

Box 1. An example of a meaningful moment (299)

She had a very aggressive cancer that was causing a lot of pain. She had a lot of pain in her heart and soul. A lot of fear. There’s no curing her cancer, her sepsis, her cancer eating into her stomach and lungs.

I can see her now, just a shell of a person, hunched over, withdrawn. The sun was streaming in the window, I was sitting, and all I did was sit there in the sun, hold her hand, and ask her questions about her life. Holding hands, looking at each other. The atmosphere of the room was so much warmer. You can physically see her opening up, in addition to her opening up emotionally. The expression on her face – there was some joy when she was recalling how she met her husband and talking about her children. Even a smile or a laugh. Seeing tears in her eyes that weren’t associated with sadness or fear. Seeing the transition of feeling like a patient, back to feeling like a person, and how much relief can come from bringing dignity back into the experience.

Something was happening between us, that made both of us emotional, and it was different from all the other times she had thanked me. …It was joy, and love, and sad, and gratitude. I felt it well up [inside me], like butterflies in my chest, and I remember wet eyes and trying not to let them fall in front of her. I didn’t cry in her room. Trying to name those emotions in the moment was hard because my body just feels the pressure, and the release is my eyeballs.

I remember feeling so much better walking out of her room later. I remember taking that feeling with me. It was like a reawakening.

(Participant 10, female, *≤*10 years in practice)

### Moments of meeting

Meaningful moments were common and easy for participants to identify: our 10 participants recounted 36 meaningful moments. The content of these moments varied widely, ranging from informing patients of a terminal diagnosis or limited prognosis to challenges in care provision, such as intractable pain, and moments of death. These moments were expressed evocatively, as embodied, with a sense of presence and emotion. Table 3 illustrates different dimensions of these experiences, exemplified through Van Manen’s existentials (van Manen 2023).

**Table 3.** A worked example of lived existentials mapped to exemplar quotes

Existential theme	Exemplar quote
**Lived time**	
Participants chose moments from the recent past to years prior, some even recalling patient encounters from when they started their careers. Regardless of when the moment had occurred chronologically, in describing the moment, tenses often moved between the past and present, suggesting that the moment was “relived” in the telling.	*It’s a kind of mindful creating of the time and space to be present. Being able to be present and not having to say very much, you know? I was able to sort of just sit for a while and be with them without too much agenda.* (Participant 2, female, 11−20 years practice)
**Lived Body**	
Participants recounted how the moment felt physically.	*It’s hard to duplicate the intensity, the emotion, the palpable relief that you can see. It was remarkable the contrast between her distress and her peace. Her voice, and the way that she connected, that was the most powerful kind of thing. That sort of experience that you can see and feel are profound.* (Participant 3, female, ≥21 years practice)
**Lived Space**	
The context of moments varied widely, from the bedside in hospitals, hospices, or home settings to encountering family members after a patient’s death. Physicians especially valued moments they shared with patients and their families in the home setting.	*It was helpful to come out of that space and into a different place where we could all sit, and yeah, have some more kind of quiet space to talk.* (Participant 1, female, ≤ 10 years practice)

#### Connecting in a moment

The moments participants described were often very brief – a matter of seconds or minutes. While in some cases the moments described were a culmination of a relationship, in others this was the first and only encounter between the patient and the physician. Participants recounted, almost surprised, how fleeting these encounters were. Regardless, the moments were described as intimate. Intimacy was characterized as a sense of connection, where connection represented the presence of patient and doctor as human beings rather than as doctor and patient.

I felt it was a person-to-person conversation rather than a patient-physician hierarchy. It felt like we both had a bit of an inside, deeper connection. …and I think that in me treating them as humans and seeing them as people and talking to him like he’s a person and not like a patient, they’re also treating me like a person too. And it is kind of weird, blurring the lines between patient-physician relationship, but it just feels so much more aligned with the way that I would want to be treated.

(Participant 8, male, ≤10 years practice)

Connecting was not about doing something in a physiological sense. In fact, participants often reflected on the limitations of their scientific training and pharmacological armamentarium. Rather, connection was “doing by being,” which was conveyed as being actively present in the moment, acknowledging existential distress and uncertainty.

Connection. You know, I think, in that moment, I instantaneously felt connected to this patient. I realised, you know, in that moment, how deeply I had gotten to know him over that period of time, despite the encounters not always being cordial.

(Participant 5, male, ≤10 years practice)

Participants expressed their interpretations of the moments as being meaningful for both the patient and the physician.

I think she really valued that human connection to tell the stories, and I really valued hearing them. It was very satisfying, mutually satisfying.

(Participant 2, female, 11–20 years practice)

#### Transformative moments

Participants ascribed the moments they recounted as having a profound impact on their personal and professional lives. While many of the moments were emotionally upsetting, participants reflected on their value.

Yeah, it’s caused me to open up to a wider perspective on what a good death is for a particular person, and what looks good to us, may not be what another person wants for themself or wishes for themself.

(Participant 5, male, ≤10 years practice)

For many, the moments carried a personal significance in relation to their identity as doctors and as people:

There’s a certain intimacy in terms of what they share with us, and what we observe, and what we’re able to be a part of. This has allowed me to learn about life and death. It has given me a richness and an appreciation for life that I wouldn’t have had otherwise had.

(Participant 7, male, ≥21 years practice)

Participants recounted how these moments affirmed their commitment to palliative care and were a source of renewal and energy.

I remember feeling so much better walking out of Jerry’s room later. I remember taking that feeling with me. The reminder that this is why I chose palliative care, that helps buffer against tough days to come. I left the room thinking, I’m going to do that way more often. …I left, remembering this is why I chose palliative care: because I can sit back and learn about my patients. It makes me feel good, and makes my patients feel good.

(Participant 10, female, ≤ 10 years practice)

## Discussion

### Main findings

At times of low morale, disillusionment, and workforce burnout, this study shares how physicians caring for dying patients were able to find meaning and inspiration from brief moments of significance that had a lasting impact. These moments were characterized as experiencing a sense of connection with patients and inter-personal revelation, described as intimate and mutual. These moments fueled participants’ commitment to their profession.

### What this study adds

To elucidate theoretical findings in more depth, we draw on ideas from hermeneutic philosophical literature to consider how this can expand our understanding of meaning-making in the praxis of palliative care.

#### Suffering and time

Suffering disrupts time: the present is not what the past was supposed to foreshadow (Egnew 2009). In palliative care, the constant companion of “being toward death” (Heidegger 2010) assumes a new immediacy. The existential crisis of being disrupts the patient’s everyday world of know-how, of being and doing, to reformulate time, bringing the temporality of one’s existence to the fore (Heidegger 2010; Hyde 2005). A central component of moments of meaning was an exchange between patient and physician, which acknowledged this predicament. Contrary to the assumption that such acknowledgment required a lot of time, the moments described by participants were typically brief – a matter of seconds, minutes at most. In these accounts, we propose, time was not experienced chronistically, as in clock time of minutes, hours, and so on, but assumed a different qualitative dimension, experienced as *kairos*: a critical *kairiotic* moment presents those experiencing it with an opportunity to make a difference and to do the right thing. Time slowed down, expanded to become an experience outside manufactured time, to survive “inside” time; suspended, coalescing past and present, projecting into future patient care. In his seminal text “Being and Time” (Heidegger 2010) Heidegger introduced the idea of *Mitsein*, of “being with.” In encounters of shared time, there is an awareness of the other’s time, or lack thereof, which creates a new experience of time (McMullin 2012). In moments of meaning, doctor and patient opened to each other’s time to create new possibilities of seeing each other.

#### Acknowledging existential moments

Moments of meaning were intimate. Etymologically, intimate means “to make known.” In *kairiotic* moments, where the “acuity of suffering lies in the impossibility of retreat” (Levinas 1987), the physician acknowledged and bore witness to the patient’s suffering: doctor and patient were made known to each other as human beings. French philosopher Emanuel Lévinas (1906–1995) described the inter-human, an intersubjective experience where the difference between two people facilitates a recognition of the other’s immediate existence. Witnessing suffering in moments of meaning becomes a type of ethical encounter, where the face of the other – which refers not literally to the face, but rather to the experienced presence – awakens a responsibility and wish to care for them. Sharing an existential moment opened a healing connection around the commonality of human woundedness (Nouwen 1979). The ethics of the face may be seen as an absolute witnessing, meaning that human beings are there for human beings (Levinas 1979). In moments of meaning, physicians respond to the call of the other, in this instance, their patient.

#### Transformation & transcendence

Meaningful moments were deeply felt experiences that participants carried forward to inform future patient care. Transcendence describes an experience that extends or lies beyond the limits of the ordinary. In these shared moments, physicians let go of the need to “do” and “solve” to reconcile their role: as witnesses, as human beings accompanying a patient’s journey of suffering. “Suffering ceases to be suffering in some way,” Victor Frankl observed, “at the moment it finds a meaning” (Frankl 2004). In this way, the sense of being able to acknowledge existential mortality transformed moments of suffering into moments of meaning. Making sense of these moments, several participants commented on how they would seek out such moments to sustain and renew their commitment to their professions. According to Levinas, experiencing the ethics of the face does not deprive one of freedom but rather gives back uniqueness: when a person puts themselves at the disposal of the other, they are witnessing that there is something beyond and are thereby a witness of the eternal and universal. In this manner, moments of meaning become interpersonal encounters carrying an ontological significance, revealing what is important to physicians and what gives meaning to their world.

### Clinical implications

Meaningful moments were commonly experienced by our participants, to the extent that they often had multiple ones to choose from. This is an encouraging finding. Further, as participants reflected on the moment and the experience of recounting it, they often probed more deeply into the significance of the moment for themselves. The value of reflecting on transformative experiences in palliative care has been shown previously (Mota Vargas et al., 2016; Sapeta et al., 2022; Wiesner et al., 2024). Recounting moments perhaps had some cathartic value: despite experiencing emotions during the interview, participants expressed a sense of renewed insight. While our findings suggest that sharing meaningful moments may be of value, it does not tell us what approach to use.

The value of reflective practice and meaning-making to counter burnout is well recognized (Sapeta et al. 2022; Shalev et al. 2022; West et al. 2018) with various formats (individual, group, written) advocated (Clandinin and Cave 2008; Maben et al. 2021; Moniz and Ng 2024; Rabow et al. 2014). Yet, such activities are often viewed as optional extras, taking time away from already depleted physicians. Integrating such activities so that they are considered routine could afford a means to infuse physicians with a sense of purpose and build shared identities of practice. This offers an avenue for development as part of mindful practice initiatives, wellness programming, and education workshops.

### Study limitations

Our study is limited to a particular context with a group of volunteer participants. These data are limited to physicians working in palliative care and do not include other professions, such as clinical nurse specialists or extended team members working in this area, or indeed other disciplines within medicine. It is possible that the intensity of palliative care is a context that promotes such experiential encounters, and an area for future research could involve identifying moments of meaning in other disciplines. While our participants reported a sense of mutual connection, we do not know if this was the experience of patients. Furthermore, although our findings provide some insight into how palliative care physicians make meaning when caring for patients, many other motivations and drivers of meaning exist, such that our findings only give a partial insight into the richness of how we, as physicians, make sense of our worlds. In addition, our focus on existential dimensions of meaning-making does not necessarily mitigate the multitude of organizational, institutional, and administrative contributors to burnout in palliative care provision. Finally, in keeping with the hermeneutic tradition, we recognize that our interpretation is informed by our own pre-understandings and that a different group of researchers may have drawn different meanings from the data.

## Conclusion

Palliative care physicians identified many experiences of meaning in their day-to-day practice. These experiences were often momentary – a glance, a sentence or two, sitting with a patient or their family. In these moments, physicians experienced a sense of connection, where the personhood of both patient and physician was realized. Participants experienced a sense of personal transformation, being deeply moved and inspired by the privilege of sharing these moments. The meaning participants took from their experiences projected through time. These moments consolidated what mattered to participants and were carried forward as exemplars of what being a palliative care physician meant, as well as to inform the care of future patients. Reflecting on these moments may be a sustaining practice for physicians and could be explored as an inspiring part of learning for trainees, and as a therapeutic activity for physicians experiencing demoralization or burnout.

## Appendix 1: An additional example of a meaningful moment

I’ll tell you a story of a gentleman I cared for, in an inner-city hospice. He was dying. He was always cantankerous and never satisfied with his management. He was mad at the world.

He would get himself into a wheelchair – clinically unstable in respiratory distress – and he would go outside, hanging out with his friends, smoking his cigarette and occasionally crack cocaine. About 24 hours before he died, I saw him in the parking lot, slumped over in his wheelchair and lamenting about his poor symptom control and insisting that he stay out there in the cold.

I felt really helpless, I turned to him and asked – “*what more can we do to assist you in this journey?”*

He turns to me and says, *you’re already doing it. All you need to do is just listen to my story. And I’m fine; this is just my story.*

In that moment, I instantaneously felt connected to this patient. In that moment, I realized how deeply I had gotten to know him, despite the encounters not always being cordial. I’ve still gotten to appreciate his personality, his struggles, and to known him as a person, and in this moment, I saw yet another side of him.

He had accepted his suffering more than I had accepted his suffering; suffering was a way of life for himself, and that was okay, so long as he could balance it with the things that were meaningful to him. The only thing he had control over is what he did in those final days.

That sense of connection to another human being, appreciating their complexity, the uniqueness of their journey, is really meaningful for me. When you’ve exhausted all other measures, the support you provide someone as just another human being, can be so powerful.

Sometimes we don’t realize the power of that support that we’re providing, we don’t see it in ourselves.

Moments like that show you the nature of basic humanity: it isn’t about the medications, the interventions, or all the fancy things we learn. Sometimes it’s just as simple as being there, being witness to somebody else’s suffering, and supporting them in their journey, human to human. (Participant 5)
